# Screening and Antioxidant Activities Evaluation of Peptides From Abalone (*Haliotis discus hannai* Ino)

**DOI:** 10.1002/fsn3.70028

**Published:** 2025-02-09

**Authors:** Jingna Wu, Xiaoya Wang, Kun Qiao, Nan Pan, Xiaoting Chen, Zhiyu Liu, Yuanxin Chen

**Affiliations:** ^1^ Xiamen Key Laboratory of Marine Medicinal Natural Products Resources/Fujian Universities and Colleges Engineering Research Center of Marine Biopharmaceutical Resources Xiamen Medical College Xiamen P. R. China; ^2^ Fujian Fisheries Research Institute Xiamen P. R. China

**Keywords:** abalone, antioxidant peptide, Keap1–Nrf2 pathway, molecular docking, oxidative damage

## Abstract

Marine organisms are rich in antioxidant peptides; however, extracting these peptides is time‐consuming, labor‐intensive, and costly, with sequence losses leading to uncertain results. This study aimed to identify abalone‐derived antioxidant peptides with strong Keap1 binding ability and validate their antioxidative activities using a cellular oxidative damage model. We constructed an abalone‐derived peptide library comprising 363 peptides using virtual enzymatic hydrolysis techniques. Of the 98 human Keap1 protein structures available in the protein data bank database, 2FLU was selected as the receptor. Using the CDOCKER module in Discovery Studio software, molecular docking was performed with the peptide library as ligands and 2FLU as the receptor, targeting the binding site at coordinates *x*: 5.000222, *y*: 7.103889 and *z*: 5.058000. Ten abalone‐derived peptides with the strongest inhibition against Keap1–Nrf2 interaction were identified. A 2,2′‐azobis (2‐methylpropionamidine) dihydrochloride (AAPH)‐induced oxidative damage model in human umbilical vein endothelial cells (HUVECs) was used to verify the molecular docking results and identified DEDEDEDK as the most active antioxidant peptide. DEDEDEDK interferes with Keap1–Nrf2 binding, significantly reducing reactive oxygen species levels in damaged cells, increasing superoxide dismutase and catalase activities, and elevated glutathione content, indicating its potential to mitigate AAPH‐induced oxidative damage in HUVECs.

## Graphical abstract

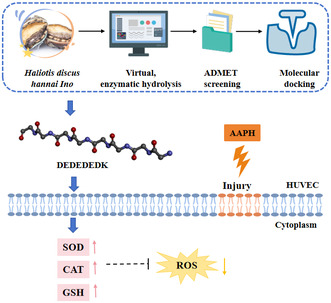

## 1 Introduction

Oxidative stress occurs because of an imbalance in the body's oxidative–antioxidative system, leading to excessive accumulation of reactive oxygen species (ROS) (Sheng et al. 2023). This can damage biomolecules, such as nucleic acids, proteins, and lipids, potentially contributing to conditions such as atherosclerosis, liver disease, cancer, and aging (Lowe et al. 2017; de Almeida et al. 2017). Enhancing the body's antioxidant capacity is crucial for maintaining health. Most commercial antioxidants are chemically synthesized and may carry certain toxic risks to humans (Jiao et al. 2020). Therefore, the development of nontoxic, effective natural antioxidants is crucial. With the increasing availability of protein resources, diverse proteases, and advancements in separation and purification technologies, more antioxidant peptides have been discovered (Chen et al. 2019; Zanutto‐Elgui et al. 2019).

Antioxidant peptides are obtained from food proteins by simulating gastrointestinal digestion using pepsin, trypsin, or chymotrypsin. The proteins are degraded, and the product is subjected to separation, purification, and sequence identification to screen for peptides with antioxidant activity (Shen et al. 2010). However, this method is time‐consuming, labor‐intensive, and costly, and peptide sequences are often lost during purification, leading to uncertain results. Computer‐aided design (CAD) can accelerate bioactive peptide development from laboratory to market. First, the amino acid sequences of the source proteins are retrieved from the National Center for Biotechnology Information, protein data bank (PDB), and UniProt databases. After obtaining the primary structures, virtual enzymatic hydrolysis is performed using online tools, such as BIOPEP, Enzyme Predictor, and PeptideRanker. With the 3D structure of the target receptor protein known, molecular docking and dynamics simulations are used to explore receptor protein and ligand molecule interactions and evaluate their binding affinity. Thus, virtual enzymatic hydrolysis and molecular docking can be used to predict and discover numerous peptides under simulated conditions and efficiently screen structurally novel bioactive peptides (Yu et al. 2018). This approach reduces the workload and costs, shortens the development cycle, and increases the success rate of identifying active peptides. Researchers have screened antioxidant peptides from numerous protein sources, including fish, sea cucumbers, and soybeans, using CAD (Wu et al. 2024).

The Keap1–Nrf2 signaling pathway is a crucial antioxidant mechanism in the body (Zanutto‐Elgui et al. 2019). Inhibiting the Keap1–Nrf2 interaction can activate this pathway, promoting the expression of genes related to antioxidant and cell‐protective proteins, thereby enhancing the body's antioxidant capacity (Zhuang et al. 2014). Nrf2, a key transcription factor that regulates cellular oxidative stress, is usually bound by Keap1 and is rapidly ubiquitinated and degraded. However, under oxidative stress, Nrf2 escapes Keap1‐mediated degradation, enters the nucleus, and binds to antioxidant response elements, activating the expression of a series of antioxidative and cell‐protective genes to enhance the body's antioxidative defense (Adelusi et al. 2020). Therefore, screening for antioxidant peptides targeting the Keap1–Nrf2 pathway is a more specific approach.

Marine organisms are important sources of antioxidant peptides due to their diversity and unique nutritional components. Various antioxidant peptides are derived from marine protein sources, such as shellfish, fish, and algae (Yuan et al. 2018). For instance, MDLFTE and WPPD isolated from the protein hydrolysate of the marine bivalve mollusk *Tergillarca granosa*, demonstrated strong scavenging activities against several radicals, including the 2,2‐diphenyl‐1‐picrylhydrazyl (DPPH) radical, hydroxy radical, superoxide anion radical, and the 2,20‐azino‐bis‐3‐ethylbenzothiazoline‐6‐sulfonic acid (ABTS) cation radical, along with potent inhibitory effects on lipid peroxidation (Yang et al. 2019). Similarly, peptides such as PELDW, WPDHW, FGYDWW, and YLHFW, derived from the protein hydrolysate of Spanish mackerel (*Scomberomorous niphonius*) muscle, exhibited high scavenging activities against the DPPH radical, hydroxyl radical, and superoxide anion radical. These peptides also dose‐dependently inhibited lipid peroxidation in the linoleic acid model system and protected plasmid DNA (pBR322DNA) from oxidative damage induced by hydrogen peroxide in the tested model systems (Zhao et al. 2019). Furthermore, 18 antioxidant peptides were purified from the swim bladder of monkfish ( *Lophius litulon* ), including YDYD, ARW, and DDGGK, which demonstrated strong radical scavenging activity, inhibition of lipid peroxidation, ferric‐reducing antioxidant power, and protective effects on oxidation‐damaged plasmid DNA and HepG2 cells (Sheng et al. 2023).

However, compared with terrestrial sources, research on marine‐derived antioxidant peptides is relatively limited, necessitating further exploration and development. Abalone, a primitive marine shellfish rich in nutrients with high medicinal value, is an excellent resource for the development of marine bioactive peptides. Therefore, this study focused on *Haliotis discus hannai* Ino and used virtual enzymatic hydrolysis technology to establish a peptide library. By focusing on the Keap1–Nrf2 antioxidative pathway and using molecular docking technology, we screened for abalone‐derived antioxidant peptides with strong Keap1 binding ability and validated their antioxidative activities using a cellular oxidative damage model, providing a theoretical basis for the research and application of antioxidant peptides.

## 2 Materials and Methods

### 2.1 Materials and Reagents

The assay kits for total superoxide dismutase (SOD, A001‐3‐1), catalase (CAT, A007‐1‐1), reduced glutathione (GSH, A006‐2‐1), and total protein quantification (bicinchoninic acid assay, BCA, A045‐4) were purchased from Nanjing Jiancheng Bioengineering Institute (China). RPMI‐1640 medium and the CCK‐8 kit were sourced from Wuhan Sanying Biotechnology Co. Ltd. (China). Serum‐free cell freezing medium was from Xian Saimei Biotechnology Co. Ltd. (China). Fetal bovine serum (FBS) was from Shanghai Feilin Biotechnology Co. Ltd. (China). The 2,2′‐azobis(2‐methylpropionamidine) dihydrochloride (AAPH) was supplied by Sigma–Aldrich (Shanghai) Trading Co. Ltd. Human umbilical vein endothelial cells (HUVECs) were obtained from Shanghai Meixuan Biotechnology Co. Ltd. (China). The peptides FGHISV, EDE, FEPETTEEVR, GEYQ, AVVESK, DTSTMGYMAAK, DASCK, FTWVSQSNHIPMEIEEDSAKPWL, DDIMEDKDNF, and DEDEDEDK were synthesized by Genscript Biotechnology Co. Ltd. (China).

### 2.2 Construction and Optimization of the Peptide Library

Amino acid sequences of the nine proteins from *H. discus hannai* Ino were retrieved from the UniProt database (https://www.uniprot.org/) and are listed in Table 1. Virtual enzymatic hydrolysis of abalone proteins was performed using the ExPASy PeptideCutter tool (http://web.expasy.org/peptidecutter/) with pepsin (pH 1.3) (EC 3.4.23.1), trypsin (EC 3.4.21.4), and chymotrypsin (EC 3.4.21.1). Single, dipeptide, and repetitive peptides were excluded, resulting in an abalone‐derived peptide library of 363 peptides (Table S1).

**TABLE 1:** *Haliotis discus hannai* Ino proteins retrieved from UniProt.

UniProt ID	Protein names	Sequence length
J9PDF7	Delta‐5 fatty acid desaturase‐2	439 AA
K9JAG6	l‐Gulonolactone oxidase, LGO, 1.1.3.8	454 AA
D0V3X9	Heat shock protein 90	728 AA
D2JPI4	Methanethiol oxidase, 1.8.3.4	49 7AA
D4NUW9	Glutathione peroxidase	222 AA
E2E9D9	Ferritin, 1.16.3.1	171 AA
E7CHE5	C‐type lysozyme	146 AA
E7CHE6	C‐type lysozyme	146 AA
I1T027	Cysteine proteases inhibitor	271 AA

Using Discovery Studio scripts, the peptide library was constructed in batches and subjected to energy minimization (Li et al. 2017). The absorption–distribution–metabolism–excretion–toxicity (ADMET) descriptor function in the Discovery Studio software was used to predict ADMET properties. Molecules with an ADMET solubility level < 5, a positive ADMETEXT CYP2D6 prediction, ADMET EXT hepatotoxic prediction, and ADMET EXT PPB prediction were removed. Additionally, different conformations of the same peptide were eliminated.

### 2.3 Establishment of the Molecular Docking Model

#### 2.3.1 Receptor Selection and Optimization

A total of 98 PDB files of human Keap1 protein were retrieved from the PDB (http://www.rcsb.org) (Table S2). Suitable receptor files for docking were screened and optimized. This included obtaining the crystal structure of the target protein, removing redundant conformations, supplementing incomplete amino acid residues, adding hydrogen atoms, and defining the receptor‐binding sites.

#### 2.3.2 Selection of the Binding Site

The original binding site was determined based on the ligand in the PDB file for Keap1. A new binding site was designed based on this reference and used as the binding site for subsequent experiments.

#### 2.3.3 Molecular Docking

The optimized abalone‐derived peptide library served as the ligand file, and optimized Keap1 was used as the receptor file. Molecular docking was performed using the newly designed binding site as the docking site via the CDOCKER module in Discovery Studio software.

### 2.4 Screening of Abalone‐Derived Antioxidant Peptides Based on the HUVEC Oxidative Damage Model

#### 2.4.1 Cytotoxicity Testing of Peptides in HUVECs

Peptide cytotoxicity in HUVECs was assessed using the CCK‐8 assay to determine cell viability (Suo et al. 2022). HUVECs were digested with 0.25% trypsin, resuspended in RPMI 1640 complete medium containing 10% FBS at a density of 5 × 10^4^ cells/mL, and seeded into 96‐well plates at 100 μL/well. Cells were incubated in a CO_2_ incubator (37°C, 5% CO_2_) for 12 h.

Control and experimental groups were established with six replicates each. After cell adhesion, 10 μL of peptide solution was added to the experimental groups at final concentrations of 3.9, 7.8, 15.6, 31.25, 62.5, 125, 250, 500, and 1000 μg/mL, whereas the control group received an equivalent volume of complete medium. After 24 h of incubation in the CO_2_ incubator, 10 μL of CCK‐8 solution was added to each well, and cells were incubated for 1 h. Optical density (OD) was measured at 450 nm using an Infinite M1000 Pro microplate reader (Tecan, Switzerland). Cell viability was calculated using the following formula:

$$\text{Cell Viability}\left(\%\right)=\frac{A_{S}-A_{O}}{A_{C}-A_{O}}\times 100\%$$

where *A*_C_ represents the OD of the control group, *A*_S_ indicates the OD of the experimental group, and *A*_O_ is the OD of the blank wells.

#### 2.4.2 Establishment of the AAPH‐Induced HUVEC Oxidative Damage Model

The oxidative damage model was established using the method described by Li et al. (2023). HUVECs were seeded into 96‐well plates at a density of 5 × 10^4^ cells/mL at 100 μL/well and incubated in a CO_2_ incubator (37°C, 5% CO_2_) for 12 h. After cell adhesion, the model group was treated with 10 μL of AAPH solution at final concentrations of 0.25, 0.5, 1, 2, 4, 6, 8, 10, and 12 mmol/L, whereas the control group received an equivalent volume of medium. After 24 h of incubation, cell viability was assessed using the method described in Section 2.4.1 to determine the optimal AAPH concentration for inducing oxidative damage in HUVECs.

#### 2.4.3 Effect of Peptides on AAPH‐Induced HUVEC Damage

The experiment included control, model, and experimental groups. HUVECs were seeded into 96‐well plates at a density of 5 × 10^4^ cells/mL at 100 μL per well, with six replicates per group. After cell adhesion, the model and experimental groups were treated with 10 μL of AAPH solution to a final concentration of 6 mmol/L, whereas the control group received an equivalent volume of medium. After 24 h, the experimental groups were treated with 10 μL of peptide solutions to final concentrations of 3.9, 7.8, 15.6, 31.25, 62.5, 125, 250, 500, and 1000 μg/mL. The control and model groups received an equivalent volume of medium. After another 24 h of incubation, cell viability was assessed using the method described in Section 2.4.1.

### 2.5 Effect of the Peptide DEDEDEDK on AAPH‐Induced HUVEC Damage

#### 2.5.1 Detection of ROS Levels

Model and experimental groups were established, and HUVECs were seeded into 96‐well plates at a density of 5 × 10^4^ cells/mL at 100 μL/well, with six replicates per group. After cell adhesion, the control group received the medium, whereas the model and experimental groups were treated with 10 μL of AAPH solution to a final concentration of 6 mmol/L. After 24 h of incubation, experimental groups were treated with 10 μL of peptide DEDEDEDK at final concentrations of 250, 500, and 1000 μg/mL. The control and model groups received an equivalent volume of the medium. After another 24 h of incubation, ROS levels were measured according to the instructions of the ROS detection kit. Cells were washed twice with PBS, treated with 10 μL of 10 μmol/L DCFH‐DA working solution, and incubated in the dark for 30 min in the CO_2_ incubator (37°C, 5% CO_2_). The cells were then washed three times with PBS, and the fluorescence intensity was measured using an Infinite M1000 Pro microplate reader (Tecan, Switzerland).

#### 2.5.2 Detection of SOD, GSH, and CAT Levels

The model and experimental groups were established, and HUVECs were seeded into 6‐well plates at a density of 5 × 10^4^ cells/mL at 2 mL/well, with three replicates per group. After cell adhesion, the model and experimental groups were treated with 2 mL of AAPH solution at a final concentration of 6 mmol/L, whereas the control group received an equivalent volume of the medium. After 24 h of incubation, experimental groups were treated with 200 μL of peptide DEDEDEDK at a final concentration of 250, 500, and 1000 μg/mL. The control and model groups received an equivalent volume of the medium. After 24 h incubation, the cells were collected, and the protein content was measured using the BCA protein assay kit. The activities of SOD, CAT, and GSH were determined using the respective assay kits.

### 2.6 Statistical Analysis

Data were expressed as mean ± standard deviation (*X* ± SD). Statistical significance was determined using Student's *t*‐tests and Duncan's multiple range test. Differences were considered significant at *p* < 0.05, highly significant at *p* < 0.01, and nonsignificant at *p* > 0.05.

## 3 Results

### 3.1 Molecular Docking

#### 3.1.1 Screening and Selection of Receptor Files

The Pox virus and zinc finger (POZ) and Kelch regions are critical areas that influence the binding of the Keap1 protein receptor to Nrf2 (Hiebert and Werner 2023). The difference is that the POZ region affects the Keap1–Nrf2 interaction through a conformational change in the key amino acid residue Cys151 (Suzuki et al. 2023), whereas the Kelch region is the specific binding site for Keap1 and Nrf2 (Cleasby et al. 2014; Wakabayashi et al. 2010). Among the 98 Keap1 protein PDB files retrieved, 4CXT, 4CXI, 4CXJ, 5DAD, 5DAF, 7EXI, 7X4W, 7X4X, 3VNH, 3VNG, 6FFM, 5GIT, 6W67, 6W68, 6W69, 6WCQ, 5NLB, 6W66, 4AP2, and 4APF contained the POZ region structure. Files 6TH3, 6TGY, 7VIJ, and 7XPY contained the structures of the NTR, IVR, and CTR regions, which did not include the Kelch region structure that binds Keap1 to Nrf2. Therefore, they were not suitable as receptor files for molecular docking experiments. The remaining 75 files contained the Kelch region structure of Keap1 and had the potential to serve as receptor files for molecular docking experiments.

Among these 75 PDB files, 65 files (including 4IFN, 6SP4, 6SP1, 5WHL, 5WHO, 5WIY, 7Q5H, 7Q6Q, 7Q6S, 7Q8R, 7Q96, 6V6Z, 1ZGK, 4IN4, 4IQK, 6TYP, 6TYM, 6HWS, 6UFO, 4L7B, 4L7C, 4L7D, 4N1B, 7XM2, 7XM3, 7XM4, 7XM5, 6Z6A, 4XMB, 7XOT, 7K2F, 7K2H, 7K2I, 7K2L, 7K2M, 7K2N, 7K2O, 7K2P, 7K2Q, 7K2R, 7K2S, 6FMP, 6FMQ, 6T7Z, 6TG8, 4IFL, 7K28, 7K29, 7K2A, 7K2B, 7K2C, 7K2D, 7K2E, 7K2K, 2FLU, 5WFV, 3ZGC, 6T7V, 6GY5, 5WG1, 7K2J, 7K2G, 4CH9, 4CHB, 5X54, 4ASC) contained their own ligand molecules (Table S3). These ligands serve as benchmarks for designing appropriate binding sites for molecular docking experiments. Therefore, compared with the remaining PDB files without ligands, they were more suitable as receptor files for molecular docking experiments.

From Table S3, 31 PDB files, including 4IFN, 6SP4, 6SP1, 5WHL, 5WHO, 5WIY, 7Q5H, 7Q6Q, 7Q6S, 7Q8R, 7Q96, 6V6Z, 1ZGK, 4IN4, 4IQK, 6TYP, 6TYM, 6HWS, 6UFO, 4L7B, 4L7C, 4L7D, 4N1B, 7XM2, 7XM3, 7XM4, 7XM5, 6Z6A, 4XMB, 7XOT, and 4ASC, had ligands that are nonpeptide organic compounds containing aromatic functional groups. Conversely, 35 PDB files, including 7K2F, 7K2H, 7K2I, 7K2L, 7K2M, 7K2N, 7K2O, 7K2P, 7K2Q, 7K2R, 7K2S, 6FMP, 6FMQ, 6T7Z, 6TG8, 4IFL, 7K28, 7K29, 7K2A, 7K2B, 7K2C, 7K2D, 7K2E, 7K2K, 2FLU, 5WFV, 3ZGC, 6T7V, 6GY5, 5WG1, 7K2J, 7K2G, 4CH9, 4CHB, and 5X54, had peptide ligands. Among these, 7K2F, 7K2H, 7K2I, 7K2L, 7K2M, 7K2N, 7K2O, 7K2P, 7K2Q, 7K2R, 7K2S, 6FMP, 6FMQ, 6T7Z, 6TG8, 4IFL, 7K28, 7K29, 7K2A, 7K2B, 7K2C, 7K2D, 7K2E, 7K2K, 2FLU, 5WFV, 3ZGC, and 6T7V contained ETGE sequence Nrf2 peptides. The binding site of Nrf2 peptides containing the ETGE (79–82) sequence with Keap1 can be considered the binding site of Keap1–Nrf2. Therefore, these PDB files are more suitable as receptor files for molecular docking than those for other compound ligands. Among these, 6T7V, 4IFL, and 2FLU were single‐chain proteins. The peptide ligand PDB files 2FLU, 6T7V, and 4IFL were specifically analyzed to select the most suitable receptor file.

**FIGURE 1:**
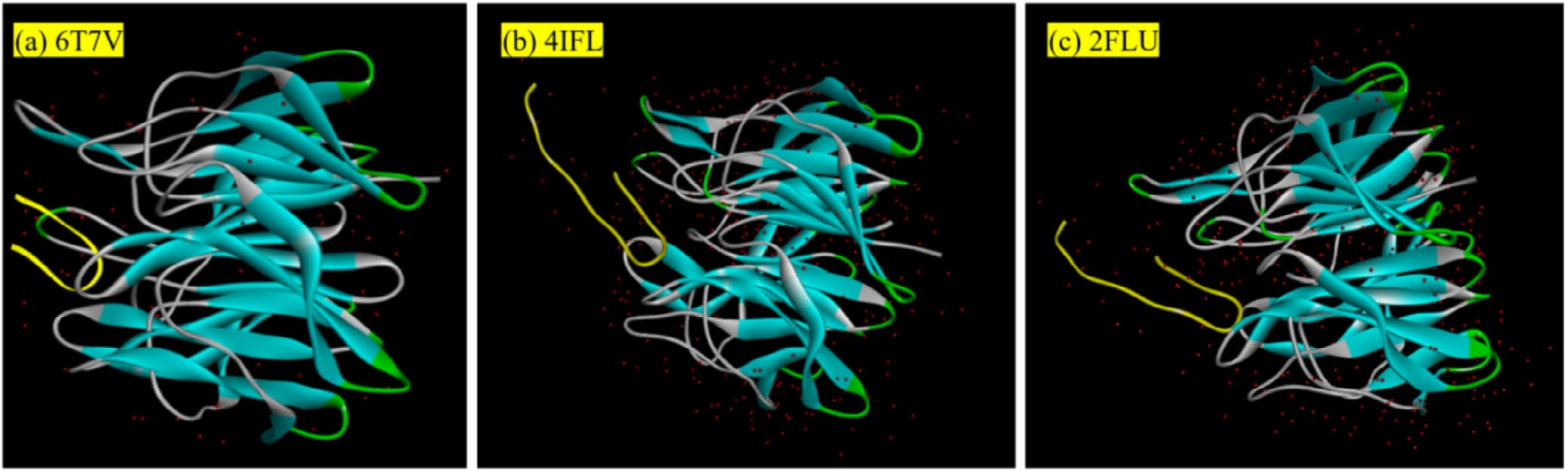
The 3D structure of the receptor–ligand complex for (a) 6T7V, (b) 4IFL, and (c) 2FLU.

Figure 1 shows the ligand–receptor complex structures of the PDB files 6T7V (a), 4IFL (b), and 2FLU (c). The ligands in 4IFL and 2FLU were the same, being 16‐mer Nrf2 (69–84) peptides containing the ETGE (79–82) sequence, whereas the ligand structure in 6T7V was a 9‐mer Nrf2 (76–84) oligopeptide. The original binding sites were determined by optimizing the ligand–receptor complex structures of the three PDB files based on the ligands in the files. After removing the original ligands, the receptors were re‐docked with their respective ligands using CDOCKER to obtain the energy scores. The ‐CDOCKER energy includes the interaction energy between the ligand and the receptor as well as the ligand's strain energy, assessing the overall binding stability of the ligand and the receptor. In contrast, the ‐CDOCKER interaction energy considers only the interaction energy between the ligand and receptor, providing a more direct reflection of the interaction capability at the binding sites. Higher values of ‐CDOCKER energy and ‐CDOCKER interaction energy indicate lower energy after the peptide binds to the Kelch domain of the Keap1 protein, suggesting a stronger binding capability (Giri et al. 2015; Jiang et al. 2014). The docking results are presented in Table 2. Energy scores for 6T7V were lower than those for 2FLU and 4IFL, indicating that 6T7V was less suitable as a receptor file for molecular docking compared with 2FLU and 4IFL. Although the ‐CDOCKER interaction energy of 2FLU was slightly lower than that of 4IFL, its ‐CDOCKER energy was slightly higher than that of 4IFL. Notably, the PDB files 2FLU and 4IFL were remarkably similar; however, they differed significantly in resolution: 2FLU had a resolution of 1.5 Å, and 4IFL had a resolution of 1.8 Å. A smaller resolution indicates a clearer structure; therefore, 2FLU is more suitable than 4IFL as the receptor file.

**TABLE 2:** CDOCKER molecular docking results of the receptor with its ligands.

PDB ID	‐CDOCKER energy	‐CDOCKER interaction energy
4IFL	178.284	138.99
2FLU	181.77	124.339
6T7V	135.899	103.814

#### 3.1.2 Docking Site Selection

**FIGURE 2:**
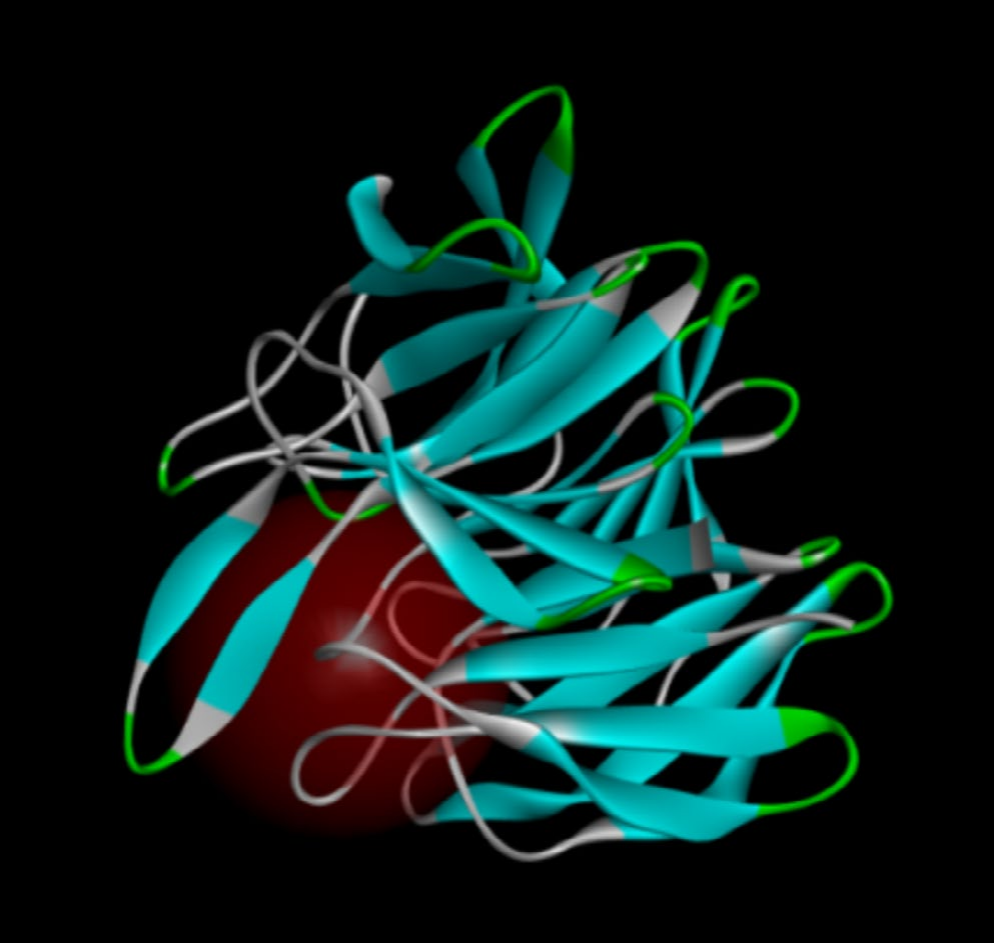
Design of 2FLU binding sites.

The ligand file provided in 2FLU was a 16‐mer Nrf2 (69–84) peptide containing the ETGE (79–82) sequence, which served as the standard for determining its original binding site. Based on this site, a new binding site was designed with a radius set to 10 Å and specific coordinates: *x*: 5.000222, *y*: 7.103889, *z*: 5.058000. The newly designed binding site is shown in Figure 2.

#### 3.1.3 Molecular Docking Analysis

In the PDB file 2FLU, the embedded ligand, 16‐mer Nrf2, formed hydrogen bonds with the amino acid residues Tyr334, Ser363, Arg380, Asn382, Arg415, Arg483, Gln530, and Ser555 in the Kelch domain of Keap1 (Lo et al. 2006). If the peptide can bind to this domain through interactions with these amino acid residues, it may interfere with the Keap1–Nrf2 interaction.

Molecular docking was performed using CDOCKER on the optimized ligand library and the receptor. The binding capability of the peptide to the Kelch domain of Keap1 was evaluated using the ‐CDOCKER interaction energy as an indicator. The top 10 peptides were selected based on their scores, and their estimated solubility, molecular weight, charge, isoelectric point, and molecular formulas are presented in Table 3.

**TABLE 3:** CDOCKER molecular docking results (Top 10).

Peptide sequence	‐CDOCKER interaction energy	Water solubility	Molecular weight (g/mol)	Charge	Isoelectric point	Molecular formula
FGHISV	119.51	Poor	658.75	0, Neutral	7.55	C_31_H_46_N_8_O_8_
EDE	113.45	Good	391.33	–3, Acidic	3.42	C_14_H_21_N_3_O_10_
FEPETTEEVR	111.97	Good	1236.29	–3, Acidic	3.82	C_53_H_81_N_13_O_21_
GEYQ	110.45	Good	495.49	–1, Acidic	3.85	C_21_H_29_N_5_O_9_
AVVESK	103.63	Good	631.72	0, Neutral	6.34	C_27_H_49_N_7_O_10_
DASCK	102.63	Good	522.58	0, Neutral	6.18	C_19_H_34_N_6_O_9_S_1_
DTSTMGYMAAK	100.38	Poor	1175.34	0, Neutral	6.23	C_48_H_78_N_12_O_18_S_2_
FTWVSQSNHIPMEIEEDSAKPWL	96.81	Poor	2745.04	–2, Acidic	4.14	C_125_H_182_N_30_O_38_S_1_
DDIMEDKDNF	96.28	Good	1241.29	−4, Acidic	3.57	C_51_H_76_N_12_O_22_S_1_
DEDEDEDK	95.75	Good	993.88	−6, Acidic	3.45	C_37_H_55_N_9_O_23_

**FIGURE 3:**
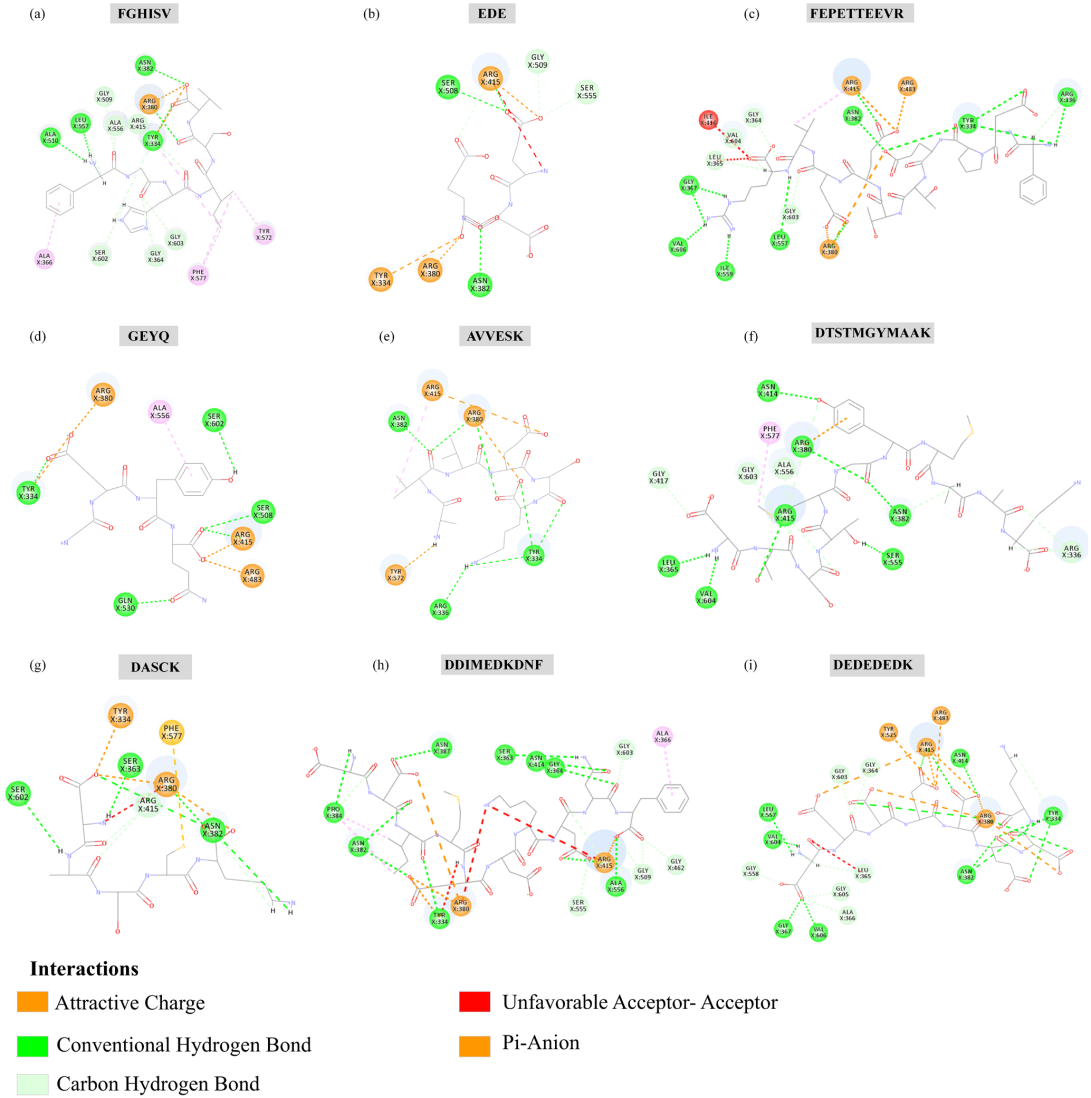
Interactions between receptor and ligand complexes for peptides (a) FGHISV, (b) EDE, (c) FEPETTEEVR, (d) GEYQ, (e) AVVESK, (f) DTSTMGYMAAK, (g) DASCK, (h) DDIMEDKDNF, and (i) DEDEDEDK.

The binding modes of the peptides to Keap1 are shown in Figure 3. FGHISV (Figure 3a) formed 13 hydrogen bonds with the amino acid residues Ala510, Leu557, Gly509, Ala556, Arg415, Tyr334, Arg380, Asn382, Ser602, Gly364, and Gly603 in 2FLU, creating a stable hydrogen bond network. The electrostatic interactions between FGHISV and the amino acid residue Arg380 in 2FLU further strengthened the stability of the complex. Additionally, the binding pocket contained several hydrophobic residues, such as Ala366, Phe577, and Tyr572, which formed strong hydrophobic interactions with 2FLU and contributed significantly to stable binding through van der Waals forces.

EDE (Figure 3b) formed six hydrogen bonds with amino acid residues Ser508, Arg415, Gly509, Ser555, and Asn382 in 2FLU, and their stable binding relied on the formation of a hydrogen bond network. The electrostatic interactions between EDE and amino acid residues Arg380, Tyr334, and Arg415 in 2FLU contributed significantly to the stability of the complex.

FEPETTEEVR (Figure 3c) formed 17 hydrogen bonds with amino acid residues Val606, Gly367, Ile559, Leu557, Gly603, Leu365, Val604, Gly364, Arg380, Asn382, Arg415, Tyr334, and Arg336 in 2FLU. This formation was crucial for the stable binding of FEPETTEEVR to 2FLU. Moreover, electrostatic interactions between FEPETTEEVR and amino acid residues Arg380, Arg483, and Arg415 in 2FLU significantly contributed to the stability of the complex.

GEYQ (Figure 3d) formed five hydrogen bonds with amino acid residues Tyr334, Gln530, Ser508, Ser602, and Arg415 in 2FLU, and their stability depended on the formation of a hydrogen bond network. In addition, GEYQ exhibited electrostatic interactions with amino acid residues Arg380, Arg483, and Arg415 in 2FLU, providing important electrostatic contributions to the stability of the complex. The binding pocket contained several strong hydrophobic residues, including Ala556, which formed strong hydrophobic interactions with 2FLU, further enhancing complex stability via van der Waals forces.

AVVESK (Figure 3e) formed seven hydrogen bonds with Asn382, Arg380, Tyr334, and Arg336 residues in 2FLU, with their stable binding relying on the formation of a hydrogen‐bond network. Simultaneously, the electrostatic interactions between AVVESK and amino acid residues Arg380, Tyr572, and Arg415 in 2FLU significantly contributed to the binding of the complex.

DTSTMGYMAAK (Figure 3f) formed 16 hydrogen bonds with amino acid residues Asn414, Arg380, Ala556, Gly603, Arg415, Gly417, Leu365, Val604, Asn382, Ser555, and Arg336 in 2FLU, and the formation of a hydrogen bond network was crucial for the stable binding of DTSTMGYMAAK to 2FLU. Furthermore, the binding pocket contained several strongly hydrophobic residues, such as Phe577, which enhanced complex stability through hydrophobic interactions via van der Waals forces.

DASCK (Figure 3g) formed eight hydrogen bonds with amino acid residues Ser602, Ser363, Arg415, Asn382, and Arg380 in 2FLU, maintaining their stable binding through the formation of a hydrogen bond network. Additionally, DASCK exhibited electrostatic interactions with amino acid residues Arg380 and Tyr334 in 2FLU, providing significant electrostatic contributions to the binding of the complex.

DDIMEDKDNF (Figure 3h) formed 18 hydrogen bonds with amino acid residues Pro384, Asn382, Tyr334, Asn387, Ser363, Asn414, Gly364, Gly603, Ala556, Gly509, Gly462, Ser555, and Arg415 in 2FLU, and their stable binding is dependent on the formation of the hydrogen bond network. Furthermore, the electrostatic interactions between DDIMEDKDNF and amino acid residues Arg380 and Arg415 in 2FLU further enhanced binding stability. The binding pocket contained several strongly hydrophobic residues, including Ala366, which formed strong hydrophobic interactions with 2FLU and contributed to the stability of the complex through van der Waals forces.

DEDEDEDK (Figure 3i) formed 20 hydrogen bonds with amino acid residues Leu557, Val604, Gly558, Gly367, Val606, Ala366, Gly605, Leu365, Gly603, Gly364, Asn414, Arg415, Arg380, Tyr334, and Asn382 in 2FLU. The formation of this hydrogen‐bond network is crucial for the stable binding of DEDEDEDK to 2FLU. Additionally, DEDEDEDK interacted electrostatically with Arg380, Tyr525, Arg483, and Arg415 in 2FLU, providing significant electrostatic contributions to the binding of the complex.

Although FTWVSQSNHIPMEIEEDSAKPWL showed a high ‐CDOCKER interaction energy in the CDOCKER energy scoring, the peptide was too long to successfully perform further docking analysis after redocking.

The best docking results at the targeted binding site (*x*: 5.000222, *y*: 7.103889, *z*: 5.058000) were for FGHISV, EDE, FEPETTEEVR, GEYQ, AVVESK, DASCK, DTSTMGYMAAK, DDIMEDKDNF, and DEDEDEDK, suggesting these peptides may interfere with the interaction between Keap1 and Nrf2.

### 3.2 Screening of Abalone‐Derived Antioxidant Peptides Using the HUVEC Oxidative Damage Model

#### 3.2.1 Cytotoxic Effects of Abalone‐Derived Antioxidant Peptides in HUVECs

Cell viability is a crucial indicator of cell survival or death after exposure to toxic substances (Wang et al. 2022). The cytotoxicity results of the 10 abalone‐derived antioxidant peptides in HUVEC are presented in Table 4. As indicated in Table 4, peptides FGHISV and AVVESK at a concentration of 1000 μg/mL significantly reduced cell viability compared with the control group (*p* < 0.01, *p* < 0.05), exhibiting an inhibitory effect on cell growth. The peptides EDE, FEPETTEEVR, DASCK, DTSTMGYMAAK, and DEDEDEDK showed no toxicity to HUVECs within the concentration range of 3.9–1000 μg/mL; however, EDE, FEPETTEEVR, DASCK, and DEDEDEDK promoted cell growth. The peptide GEYQ significantly reduced cell viability at concentrations of 3.9–62.5 μg/mL compared with the control group (*p* < 0.01), showing a clear inhibitory effect, whereas at 125–1000 μg/mL, there was no significant difference in cell viability between the peptide treatment group and normal cells (*p* > 0.05), indicating that it neither promoted proliferation nor inhibited growth of HUVECs. Peptide FTWVSQSNHIPMEIEEDSAKPWL at concentrations of 31.25–1000 μg/mL showed significant differences in HUVEC viability compared with the control group (*p* < 0.01), indicating a clear inhibitory effect. At 500 and 1000 μg/mL, the peptide DDIMEDKDNF reduced HUVEC viability to 89.96 ± 3.31 and 76.11 ± 4.35, respectively, significantly lower than normal cells (*p* < 0.01), exhibiting a clear inhibitory effect.

**TABLE 4:** Effects of different concentrations of peptides on the viability of human umbilical vein endothelial cells (HUVECs).

Peptides (μg/mL)	FGHISV	EDE	FEPETTEEVR	GEYQ	AVVESK	DASCK	DTSTMGYMAAK	FTWVSQSNHIPMEIEEDSAKPWL	DDIMEDKDNF	DEDEDEDK
Control group	100.00 ± 6.91	100.00 ± 4.78	100.00 ± 8.56	100.40 ± 7.49	100.44 ± 13.7	100.00 ± 17.0	100.00 ± 4.84	100.00 ± 3.53	100.00 ± 1.25	100.00 ± 8.23
3.9	124.26 ± 3.55**	139.91 ± 8.20**	92.19 ± 4.38	87.39 ± 4.95**	106.41 ± 12.6	124.13 ± 8.19**	110.60 ± 10.5	94.07 ± 10.9	99.78 ± 2.94	110.45 ± 8.96*
7.8	125.60 ± 4.47**	141.74 ± 6.16**	99.96 ± 5.44	88.04 ± 3.14**	107.16 ± 12.7**	122.56 ± 2.33**	107.97 ± 4.46	93.54 ± 6.15	99.91 ± 1.22	109.92 ± 9.11*
15.6	120.73 ± 3.68**	142.33 ± 4.83**	115.29 ± 9.07**	87.75 ± 3.22**	110.41 ± 10.4**	117.94 ± 12.5**	105.01 ± 15.50	91.64 ± 7.06	98.93 ± 1.93	109.45 ± 5.00*
31.25	118.42 ± 3.96**	142.41 ± 5.54**	123.95 ± 4.82**	86.3 ± 7.41**	117.69 ± 8.11**	112.37 ± 2.23*	110.68 ± 5.72	85.27 ± 7.72**	97.39 ± 1.34	108.9 ± 6.63*
62.5	111.91 ± 6.69**	142.86 ± 8.25**	128.06 ± 7.04**	91.15 ± 4.92	119.02 ± 13.5**	111.87 ± 5.97*	102.30 ± 13.90	84.82 ± 7.23**	99.38 ± 1.67	108.51 ± 7.14
125	111.60 ± 8.49**	144.45 ± 3.11**	132.09 ± 6.84**	96.50 ± 8.76	114.07 ± 11.3**	108.32 ± 8.50	98.71 ± 5.85	83.82 ± 4.36**	97.64 ± 3.32	107.27 ± 4.14
250	92.70 ± 3.19	144.81 ± 7.20**	134.03 ± 11.4**	96.70 ± 10.8	108.29 ± 15.6**	106.05 ± 2.86	93.79 ± 4.76	83.35 ± 4.63**	98.43 ± 1.31	106.79 ± 3.78
500	96.81 ± 6.18	148.55 ± 6.77**	113.58 ± 3.62*	98.33 ± 8.84	108.61 ± 8.36**	105.34 ± 4.22	93.16 ± 9.17	80.98 ± 9.58**	89.96 ± 3.31**	105.78 ± 3.79
1000	91.46 ± 3.30*	153.44 ± 11.8**	94.74 ± 5.30	98.97 ± 6.58	81.58 ± 7.42**	103.46 ± 429	94.16 ± 7.65	81.21 ± 8.60**	76.11 ± 4.35**	105.49 ± 6.27

*p < 0.05, **p < 0.01 versus control group.

High‐concentration peptides have been reported to influence cell infiltration pressure through their charge, secondary chemical bonds, and electrostatic interactions, thereby disrupting cellular metabolism and reducing cell viability (Sierra and Viñas 2021). Therefore, the study selected concentrations of 3.9, 7.8, 15.6, 31.25, 62.5, 125, 250, 500, and 1000 μg/mL as working concentrations for EDE, FEPETTEEVR, DASCK, DTSTMGYMAAK, and DEDEDEDK; 3.9, 7.8, 15.6, 31.25, 62.5, 125, 250, and 500 μg/mL for FGHISV and AVVESK; and 3.9, 7.8, 15.6, 31.25, 62.5, 125, and 250 μg/mL for DDIMEDKDNF.

#### 3.2.2 Effects of AAPH on Cell Viability

**FIGURE 4:**
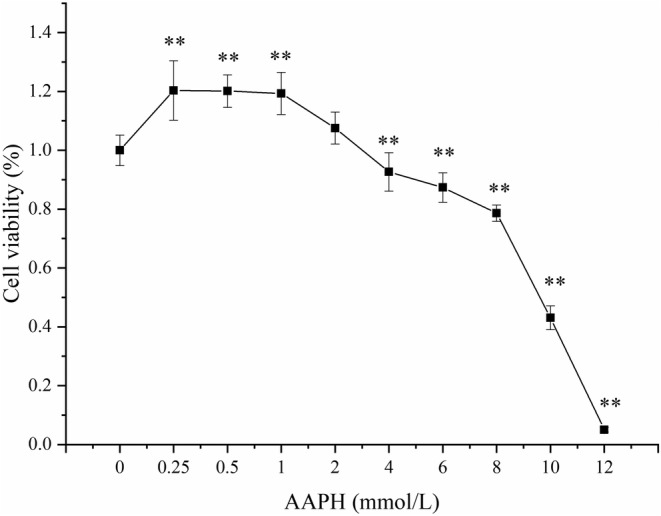
Effects of different concentrations of AAPH on HUVEC activity. **p < 0.01 versus the control group.

The results of the AAPH‐induced oxidative damage model in HUVECs are shown in Figure 4. After 24 h of treatment with various concentrations of AAPH (0.25, 0.5, 1, and 2 mmol/L), the cell viability was significantly higher than the control level of 100% (*p* < 0.01), indicating that these concentrations did not cause damage to the HUVECs and may have a promoting effect. The increased cell viability following AAPH treatment may be attributed to cellular stress responses. AAPH, as an oxidant, triggers oxidative stress in cells, but cells can activate various defense mechanisms in response to moderate levels of oxidative stress. For instance, cells may upregulate the expression of antioxidant enzymes such as SOD and GSH peroxidase, thereby enhancing their antioxidant capacity and potentially promoting cell proliferation and viability. Additionally, AAPH may influence cellular signaling pathways, such as the activation of the PI3K/Akt pathway, which promotes cell survival and proliferation (Li et al. 2016). Similar observations have been reported in other studies. For example, it has been shown that cell viability can increase after treatment with certain concentrations of hydrogen peroxide due to cellular stress adaptation mechanisms (Anasooya Shaji et al. 2019). When the final concentrations of AAPH were 4, 6, and 8 mmol/L, the cell survival rates were significantly lower than those of the control group, with differences reaching an extremely significant level (*p* < 0.01); the survival rates were 92.64% ± 0.06%, 87.35% ± 0.05%, and 78.64% ± 0.03%, respectively. However, after treatment with AAPH at final concentrations of 10 and 12 mmol/L for 24 h, the cell survival rates significantly decreased (*p* < 0.01) to 43.10% ± 0.04% and 5.07% ± 0.01%, respectively. This may be due to excessive oxidative damage within the cells, which directly leads to apoptosis.

A high cell survival rate did not allow the establishment of a clear oxidative damage model, whereas a low survival rate led to irreversible damage; both are unfavorable for oxidative stress model construction (Zhang et al. 2016). Therefore, 6 mmol/L was selected as the optimal working concentration for the AAPH‐induced damage model.

#### 3.2.3 Effects of Abalone‐Derived Antioxidant Peptides on AAPH‐Induced Cell Viability Damage

The protective effects of the abalone antioxidant peptides against oxidative stress damage in HUVECs are shown in Table 5. At different concentrations, the groups treated with peptides FGHISV, EDE, and DTSTMGYMAAK did not show significant differences in cell viability compared with the model group (*p* > 0.05). When the concentration of peptides FEPETTEEVR, AVVESK, and DASCK were in the range of 3.9–250 μg/mL, there was no significant difference in cell viability compared with the model group (*p* > 0.05). However, at 500 μg/mL for peptides FEPETTEEVR and AVVESK and at 500 and 1000 μg/mL for peptide DASCK, significant differences in cell viability were observed compared with the model group (*p* < 0.05 or *p* < 0.01). Peptides DDIMEDKDNF and DEDEDEDK showed no significant differences in cell viability compared with the model group at working concentrations of 3.9–125 μg/mL (*p* > 0.05). However, at working concentrations of 125 and 250 μg/mL for peptide DDIMEDKDNF and at 125, 250, 500, and 1000 μg/mL for peptide DEDEDEDK, significant differences in cell viability were observed compared with the model group (*p* < 0.05 or *p* < 0.01). For subsequent experiments, a concentration gradient of 250, 500, and 1000 μg/mL was selected for peptide DEDEDEDK.

**TABLE 5:** Effects of different concentrations of peptides on the activity of HUVECs damaged by AAPH.

Peptides (μg/mL)	FGHISV	EDE	FEPETTEEVR	AVVESK	DASCK	DTSTMGYMAAK	DDIMEDKDNF	DEDEDEDK
0	100.00 ± 6.36	100.00 ± 7.01	99.99 ± 10.60	100.00 ± 2.40	100.00 ± 5.39	100.00 ± 3.23	100.00 ± 5.70	100.00 ± 3.92
Model group	73.60 ± 4.79	51.25 ± 2.54	73.19 ± 3.47	61.44 ± 1.97	62.42 ± 0.77	67.93 ± 2.21	55.78 ± 3.21	59.22 ± 2.01
3.9	73.69 ± 6.47	56.53 ± 4.72	71.43 ± 8.29	62.26 ± 4.04	61.21 ± 3.01	69.47 ± 3.84	58.40 ± 4.58	58.12 ± 2.10
7.8	74.32 ± 2.98	54.03 ± 4.19	69.91 ± 3.67	60.92 ± 2.77	59.34 ± 5.21	67.22 ± 2.03	58.97 ± 6.46	57.24 ± 4.40
15.6	74.52 ± 6.35	54.92 ± 3.84	69.39 ± 8.10	61.37 ± 3.73	57.82 ± 3.91	65.15 ± 3.08	59.38 ± 3.29	60.28 ± 3.55
31.25	78.58 ± 2.81	56.04 ± 1.52	70.63 ± 8.17	61.53 ± 4.76	56.08 ± 1.92	69.17 ± 5.69	62.13 ± 5.45	61.24 ± 2.61
62.5	69.47 ± 5.53	53.87 ± 5.33	76.70 ± 7.64	60.51 ± 3.87	57.21 ± 2.96	67.59 ± 4.85	62.81 ± 5.00	63.85 ± 4.59
125	71.47 ± 4.23	53.83 ± 2.80	80.56 ± 8.80	65.05 ± 3.54	60.02 ± 3.78	71.54 ± 4.35	65.71 ± 4.72**	64.23 ± 3.15*
250	71.89 ± 3.35	52.48 ± 2.37	82.52 ± 10.27	67.29 ± 2.58	60.36 ± 3.56	—	71.6 ± 2.62**	68.2 ± 3.80**
500	78.80 ± 5.02	55.66 ± 4.77	88.59 ± 9.75**	74.04 ± 2.52**	71.66 ± 6.17**	—	—	68.4 ± 2.58**
1000	—	56.11 ± 4.87	—	—	72.67 ± 5.00**	—	—	76.89 ± 3.84**

*p < 0.05, **p < 0.01 versus the model group.

#### 3.2.4 DEDEDEDK Reduced AAPH‐Induced Oxidative Stress in HUVECs

**FIGURE 5:**
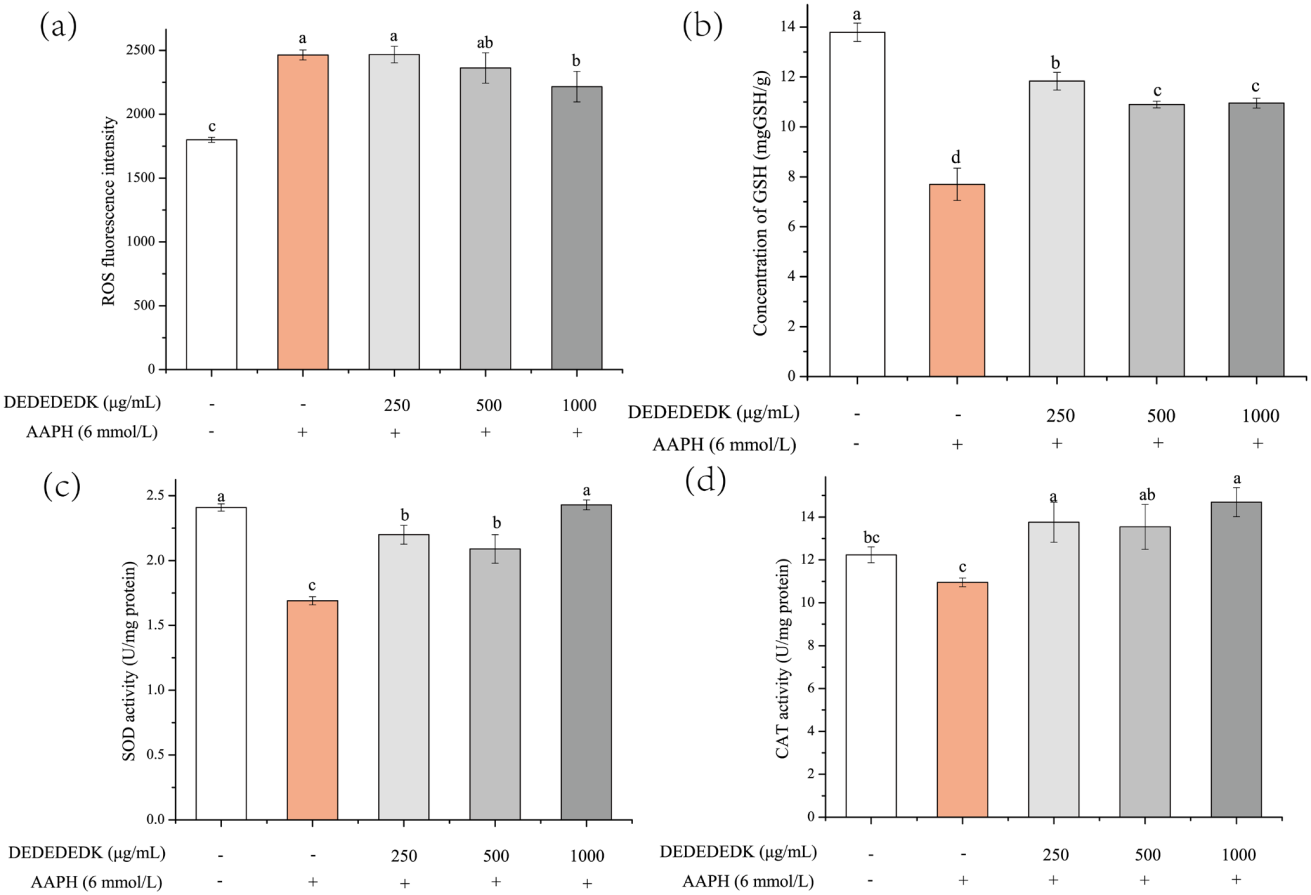
Effects of different concentrations of the peptide DEDEDEDK on oxidative stress levels in AAPH‐damaged HUVECs: (a) ROS content. (b) GSH content. (c) SOD activity. (d) CAT content. Different lower‐case letters represent significant differences (*p* < 0.05).

As shown in Figure 5a, compared with the normal control group, the model group showed a significant increase in ROS content (*p* < 0.05), indicating that AAPH treatment induced oxidative stress in HUVECs, leading to excessive ROS production. Compared with the model group, there was no significant change in ROS levels in the DEDEDEDK (250 and 500 μg/mL) treatment groups (*p* > 0.05), whereas the ROS content in the DEDEDEDK (1000 μg/mL) treatment group significantly reduced (*p* < 0.05), suggesting that high concentrations of DEDEDEDK can lower the ROS levels generated by AAPH treatment.

Figure 5b shows that the GSH content in the normal control group is 13.79 ± 0.36 mg GSH/g, whereas in the model group, the content is significantly reduced (*p* < 0.05). After adding 250, 500, and 1000 μg/mL of DEDEDEDK, the GSH content in the cells significantly increased (*p* < 0.05). However, GSH levels decrease or remain stable as peptide concentration increases. At the onset of peptide treatment, GSH levels may rise due to the activation of various cellular pathways, such as the Nrf2 pathway, which promotes GSH synthesis. As GSH levels reach a certain threshold, they can allosterically inhibit the activity of γ‐glutamylcysteine synthetase (γ‐GCS), a key enzyme in GSH synthesis. This feedback inhibition is a regulatory mechanism that helps cells maintain GSH levels within a physiologically optimal range. As peptide concentration increases and GSH levels initially surge, the inhibitory effect on γ‐GCS becomes more pronounced. This results in a decrease or stabilization of GSH production, preventing excessive accumulation of GSH and thus stabilizing or reducing GSH levels (Huang et al. 2000; Lushchak 2012).

As shown in Figure 5c,d, the SOD and CAT activities in the normal control group were 2.41 ± 0.02 and 12.24 ± 0.37 U/mg, respectively, whereas in the model group, they were 1.69 ± 0.03 and 10.95 ± 0.21 U/mg, indicating a significant difference (*p* < 0.05). After adding 250, 500, and 1000 μg/mL of DEDEDEDK, the SOD and CAT activities in the cells increased significantly (*p* < 0.05).

## 4 Discussion

Molecular docking technology was used to screen for antioxidant peptides derived from abalone that inhibited the Keap1–Nrf2 interaction. First, the proteins and their amino acid sequences were identified from the UniProt database. Virtual enzymatic digestion of abalone‐derived proteins was performed using the ExPASy PeptideCutter, which resulted in a peptide library comprising 363 small peptides. From the PDB, 98 structures reflecting the human Keap1 protein receptor were screened, with their ligands as peptides containing the ETGE sequence. Further analysis was conducted to select the 2FLU file as the receptor, and the Kelch region of Keap1 was optimized. The peptide library was used as the ligand, and new binding sites were designed based on the original ligand binding sites in 2FLU (*x*: 5.000222, *y*: 7.103889, *z*: 5.058000). Molecular docking was performed using the CDOCKER program in Discovery Studio software at this binding site, ultimately identifying the top 10 small peptides with the strongest ability to inhibit the Keap1–Nrf2 interaction: FGHISV, EDE, FEPETTEEVR, GEYQ, AVVESK, DASCK, DTSTMGYMAAK, FTWVSQSNHIPMEIEE‐DSAKPWL, DDIMEDKDNF, and DEDEDEDK.

However, because this screening experiment was performed using computer simulations, the ability to inhibit the Keap1–Nrf2 interaction in a real environment is unknown. Additionally, molecular docking tests have high false‐positive and low false‐negative rates (Magesh et al. 2012). Therefore, after the initial screening of the target ligands through molecular docking, it was essential to use other noncomputational methods to re‐screen these small‐molecule ligands to verify that they have a strong binding ability to the receptor under real conditions. Thus, we re‐screened the abalone‐derived antioxidant peptides using a HUVEC oxidative damage model. Before investigating the protective effects of the antioxidant peptides, we assessed the cytotoxic effects of the 10 abalone‐derived antioxidant peptides on HUVECs. The results indicated that EDE, FEPETTEEVR, DASCK, DTSTMGYMAAK, and DEDEDEDK exhibited no cytotoxicity at concentrations of 3.9–1000 μg/mL. FGHISV and AVVESK showed no cytotoxicity at concentrations of 3.9–500 μg/mL, and DDIMEDKDNF was nontoxic at 3.9–250 μg/mL. Conversely, GEYQ and FTWVSQSNHIPMEIEEDSAKPWL showed significant inhibitory effects on cells at concentrations 3.9–62.5 and 31.25–1000 μg/mL, respectively, so they were excluded from further study.

Subsequently, HUVECs were subjected to AAPH‐induced oxidative stress to study the effects of the eight remaining small peptides on AAPH‐induced apoptosis. AAPH, a free radical‐generating agent, induces cellular oxidative stress by initiating the production of oxygen free radicals and is widely used to establish oxidative stress models (Zhao et al. 2018). The results showed that HUVECs displayed signs of damage compared with normal cells after AAPH induction, and as the concentration of AAPH increased, cell viability decreased. A concentration of 6 mmol/L AAPH was identified as the optimal concentration for inducing HUVEC damage. Further experiments demonstrated that all eight peptides, within their respective suitable concentration ranges, resulted in resistance to AAPH‐induced cell damage and exhibited antioxidant effects, among which the peptide DEDEDEDK showed particularly good efficacy. Therefore, DEDEDEDK was selected for further analysis.

Oxidative stress is an imbalance between the oxidative and antioxidant systems and is reflected through various biochemical indicators of the degree of damage to the antioxidant defense system (Huang et al. 2019). ROS are by‐products of normal oxidative metabolism, and excessive ROS production or a compromised antioxidant defense system disrupts the balance between ROS production and elimination, leading to oxidative stress (Chaudhari et al. 2023). The results showed that AAPH treatment increased ROS levels in HUVEC cells, whereas DEDEDEDK treatment significantly downregulated ROS production. Other food‐derived peptides have also been reported as effective ROS scavengers. For instance, ADWGGPLPH from wheat germ significantly reduced ROS production in high glucose‐induced vascular smooth muscle cells (Wang et al. 2020), and WVSPLAGRT and IGFLIIWV from hemp seed hydrolysate effectively reduced hydrogen peroxide‐induced ROS levels in HepG2 cells (Bollati et al. 2022). Antioxidant enzymes such as SOD, CAT, and GSH‐Px play essential roles in antioxidant activity and are crucial for evaluating antioxidant capacity (Ma 2013). The results indicated that AAPH treatment significantly decreased the activity of SOD and CAT, as well as the content of GSH, in HUVEC cells. However, cells treated with DEDEDEDK showed significantly increased SOD and CAT activity and GSH content, although the concentration dependence was not pronounced. Furthermore, peptides such as DCN, KVVA, VCWN, WIKK, ACF, and KY, extracted from white liquor, upregulated AAPH‐induced SOD, CAT, and GSH‐Px activity in HepG 2 cells in a dose‐dependent manner (Hou et al. 2022).

In conclusion, DEDEDEDK appears to mitigate oxidative damage in HUVEC cells induced by AAPH, exhibiting antioxidant defense effects. However, whether DEDEDEDK enhances endogenous antioxidant enzyme activity by activating the Nrf2‐keap1‐ARE signaling pathway, thereby reducing cellular damage caused by oxidative stress, requires further validation in future studies.

## Author Contributions

**Jingna Wu:** conceptualization (equal), data curation (equal), funding acquisition (equal), investigation (equal), methodology (equal), writing – original draft (equal), writing – review and editing (equal). **Xiaoya Wang:** conceptualization (equal), data curation (equal), investigation (equal), methodology (equal), writing – original draft (equal). **Kun Qiao:** funding acquisition (equal), investigation (equal), methodology (equal), writing – review and editing (equal). **Nan Pan:** investigation (equal), methodology (equal), writing – original draft (equal), writing – review and editing (equal). **Xiaoting Chen:** formal analysis (equal), methodology (equal), writing – original draft (equal), writing – review and editing (equal). **Zhiyu Liu:** writing – review and editing (equal). **Yuanxin Chen:** writing – review and editing (equal).

## Ethics Statement

This study does not involve any human or animal testing.

## Consent

The authors have nothing to report.

## Conflicts of Interest

The authors declare no conflicts of interest.

## Supporting information


Tables S1–S3

## Data Availability

The data supporting the findings of this study are available on request from the corresponding author.
