# Supplementary material for: Screening and Antioxidant Activities Evaluation of Peptides From Abalone (Haliotis discus hannai Ino)
Source: Food Sci Nutr. 2025 Feb 9;13(2):e70028. doi: 10.1002/fsn3.70028 (PMC11807725; doi:10.1002/fsn3.70028)
Supplement: Supplementary file 1 — Tables S1–S3 [file FSN3-13-e70028-s001.docx]

**Table S1 Abalone-derived peptide library of 363 peptides**

| **Num.** | **Peptide** | **Num.** | **Peptide** |
| --- | --- | --- | --- |
| 1 | AAQK | 37 | DGSD |
| 2 | ACTTDYMIS | 38 | DGTR |
| 3 | ADADK | 39 | DHETL |
| 4 | ADIVR | 40 | DHK |
| 5 | ADL | 41 | DPDVR |
| 6 | AEK | 42 | DQV |
| 7 | AESS | 43 | DRPDY |
| 8 | AHEIR | 44 | DSVQNSA |
| 9 | AIR | 45 | DTSTMGYMAAK |
| 10 | APFDM | 46 | DVGGRPHWAK |
| 11 | AQA | 47 | DVMQSA |
| 12 | AQEK | 48 | DVR |
| 13 | AQF | 49 | DYVSR |
| 14 | ASE | 50 | EDE |
| 15 | ASG | 51 | EDEEK |
| 16 | ATAEK | 52 | EDEENEEKPK |
| 17 | ATT | 53 | EDF |
| 18 | AVAMVAVDGR | 54 | EDPTSHANR |
| 19 | AVDYG | 55 | EDQVEY |
| 20 | AVG | 56 | EEPCVK |
| 21 | AVK | 57 | EER |
| 22 | AVVESK | 58 | EETK |
| 23 | AWWD | 59 | EFAEK |
| 24 | CAAR | 60 | EGDK |
| 25 | CDL | 61 | EGL |
| 26 | CME | 62 | EHGGWG |
| 27 | CTAVSK | 63 | EHR |
| 28 | DACKD | 64 | EIF |
| 29 | DASCK | 65 | EIGDVYWN |
| 30 | DDAPDWVCMA | 66 | EIL |
| 31 | DDIMEDKDNF | 67 | EIVK |
| 32 | DDK | 68 | EML |
| 33 | DDVA | 69 | EMTQHTCTQVTK |
| 34 | DEDEDEDK | 70 | ENK |
| 35 | DEK | 71 | ENQK |
| 36 | DGL | 72 | ESLDPNGM |
| **Num.** | **Peptide** | **Num.** | **Peptide** |
| 73 | ETEVER | 108 | GIGSTIIYY |
| 74 | ETS | 109 | GIHEDSTNR |
| 75 | EVQPIDVRPDIEA | 110 | GII |
| 76 | EVSDDEEEEEK | 111 | GITDCPASNSMPQR |
| 77 | EYDGK | 112 | GLF |
| 78 | EYMAYAVMAY | 113 | GPGYATP |
| 79 | FEPETTEEVR | 114 | GPR |
| 80 | FGHISV | 115 | GSDITVTHDPEGQGQPDPVF |
| 81 | FGQSPSF | 116 | GSW |
| 82 | FHNDL | 117 | GTGWIPYI |
| 83 | FKPNA | 118 | GTIAK |
| 84 | FMEA | 119 | GTR |
| 85 | FNK | 120 | GTVSE |
| 86 | FRPSN | 121 | GVG |
| 87 | FSMDDVK | 122 | GVVDSEDL |
| 88 | FSVEGQ | 123 | GWG |
| 89 | FTMGVTK | 124 | HATCNVEQS |
| 90 | FTWVSQSNHIPMEIEEDSAKPWL | 125 | HGDIR |
| 91 | FWF | 126 | HHDK |
| 92 | FYDQ | 127 | HHTGWNACSSCYDDASK |
| 93 | FYPNMIK | 128 | HIFTR |
| 94 | GAPHTTHC | 129 | HLEINPDHPIVK |
| 95 | GASPQWWNHMHYQHHAKPNVMNK | 130 | HNDDEQYIWESSAGGS |
| 96 | GDNIY | 131 | HNISYK |
| 97 | GEEGMVP | 132 | HNLHK |
| 98 | GEEVEMIK | 133 | HNVM |
| 99 | GEYQ | 134 | HSGE |
| 100 | GFEVIYMTDPIDEYCVQQ | 135 | HSP |
| 101 | GGMICK | 136 | HSQF |
| 102 | GGQGTQNGISK | 137 | HVPVVG |
| 103 | GGR | 138 | HYPAK |
| 104 | GGVEPAN | 139 | IDVNGEHQHP |
| 105 | GGVTAVDVNSAEIQEVAN | 140 | IGPPLLF |
| 106 | GHI | 141 | IGYPIK |
| 107 | GIDEDDIPTEPTAESATDEMPPLEGDEDDASR | 142 | IHR |
| **Num.** | **Peptide** | **Num.** | **Peptide** |
| 143 | IINT | 181 | LSPVHGWDACYINIIMYRP |
| 144 | IISAK | 182 | LTNDWEDH |
| 145 | IKP | 183 | LVCVTK |
| 146 | IMK | 184 | LVGADGHVK |
| 147 | IMR | 185 | LVIQDSGIGMTK |
| 148 | INDV | 186 | LVIQGEVYNITDWAR |
| 149 | IQT | 187 | LVPSINR |
| 150 | ITDII | 188 | LWTR |
| 151 | ITL | 189 | LYMPYVADE |
| 152 | IVL | 190 | LYPK |
| 153 | IVNVATYUGHTHQYNG | 191 | LYR |
| 154 | IVPDK | 192 | LYSSR |
| 155 | IVQPSEVHSK | 193 | MAAATSWTAG |
| 156 | KPPQDEWGTG | 194 | MAGDVR |
| 157 | LCM | 195 | MEEVD |
| 158 | LDAMK | 196 | MEK |
| 159 | LEESK | 197 | MGK |
| 160 | LEGGPQMTQ | 198 | MH |
| 161 | LEIR | 199 | MIDVDTVNGG |
| 162 | LEPAGPVF | 200 | MIK |
| 163 | LESHW | 201 | MMGPTTI |
| 164 | LFPTMPR | 202 | MMK |
| 165 | LGK | 203 | MNL |
| 166 | LGKPGYK | 204 | MNNM |
| 167 | LGR | 205 | MPEPQEAQMEEAEVET |
| 168 | LGSGHIMISCIGDQNGDGNSG | 206 | MPESQAR |
| 169 | LGTA | 207 | MSA |
| 170 | LHNPDTTEGYVGCA | 208 | MTCTVSVWYQAWSDPK |
| 171 | LIPEY | 209 | MTDYAGQGD |
| 172 | LISNSSDA | 210 | MTEEAGR |
| 173 | LNISR | 211 | MVEK |
| 174 | LNNNIL | 212 | MVL |
| 175 | LNPDF | 213 | MVTEK |
| 176 | LQIR | 214 | NADDITQEEYAE |
| 177 | LQPSTGGCSIQTHVTSECCK | 215 | NCSYVYHSMAWY |
| 178 | LQTV | 216 | NDK |
| 179 | LSDHVTN | 217 | NDW |
| 180 | LSE | 218 | NEE |
| **Num.** | **Peptide** | **Num.** | **Peptide** |
| 219 | NGCGHPCSDYQNSNIGDDVTCVK | 256 | QVK |
| 220 | NGK | 257 | QYDITDR |
| 221 | NLVK | 258 | QYM |
| 222 | NNIK | 259 | QYVMEWAIPR |
| 223 | NPK | 260 | RHPGGSK |
| 224 | NSN | 261 | SAWDK |
| 225 | NSNDPTL | 262 | SDK |
| 226 | NTAATNTNSGGSSDYGI | 263 | SGCTY |
| 227 | NTYVTSSDH | 264 | SGFNPQHVK |
| 228 | NWVQESR | 265 | SGSM |
| 229 | PAHF | 266 | SGTK |
| 230 | PCINSDR | 267 | SIYYITGESK |
| 231 | PCNQ | 268 | SMPYNWQHR |
| 232 | PEDEEEK | 269 | SNW |
| 233 | PGL | 270 | SPSIHK |
| 234 | PHTDDVVCYHASR | 271 | SSEWGAPNAWR |
| 235 | PNGR | 272 | SSTV |
| 236 | PQL | 273 | STVDVDPESPTYCQVIHR |
| 237 | PSHGMA | 274 | SVCEK |
| 238 | PVEVR | 275 | SVNQE |
| 239 | PVY | 276 | SVS |
| 240 | QAEIAQ | 277 | SVTEING |
| 241 | QAGADISMIGQ | 278 | SYCK |
| 242 | QCEPAYN | 279 | SYGHGAQCSSVTSSY |
| 243 | QDIK | 280 | TAPVQQAVS |
| 244 | QEPGANGTEIMNGVK | 281 | TAVV |
| 245 | QGEQNS | 282 | TDPSK |
| 246 | QHD | 283 | TGH |
| 247 | QIEHH | 284 | TGL |
| 248 | QIL | 285 | TIK |
| 249 | QINSYWNCDPQDGR | 286 | TKP |
| 250 | QINV | 287 | TNWATTYSCRPEL |
| 251 | QIVSGINYI | 288 | TQICTVK |
| 252 | QNGDK | 289 | TQK |
| 253 | QNYHVNSEAGVNK | 290 | TQMCEVR |
| 254 | QQK | 291 | TQVVAGVNYV |
| 255 | QQSK | 292 | TTNT |
| **Num.** | **Peptide** | **Num.** | **Peptide** |
| 293 | TVEG | 330 | VVNAR |
| 294 | TVT | 331 | VVVSNR |
| 295 | TVAGVISTGTHGTGANYGTISSYVVAMEIMTASGEVIEVSAEK | 332 | VWSQPWL |
| 296 | VACQVPAK | 333 | VWYQSWTNTK |
| 297 | VADSNTDPQMMD | 334 | VYIPCIVPEK |
| 298 | VAER | 335 | VYIVDTGTYPR |
| 299 | VDHVTQQVVAGMK | 336 | WDM |
| 300 | VDR | 337 | WDSEK |
| 301 | VDTAS | 338 | WENSPAPDYGYD |
| 302 | VED | 339 | WYQPR |
| 303 | VEDT | 340 | WYSTYNA |
| 304 | VEE | 341 | YEPK |
| 305 | VEK | 342 | YES |
| 306 | VER | 343 | YFF |
| 307 | VESV | 344 | YGHSIHVWDWTTHER |
| 308 | VFIMDNCED | 345 | YIPML |
| 309 | VGPGL | 346 | YLKPIHIGS |
| 310 | VGQV | 347 | YMK |
| 311 | VGYYI | 348 | YNK |
| 312 | VIGYGHSPSD | 349 | YPGGDCTSDIW |
| 313 | VIR | 350 | YQL |
| 314 | VISHYAGQDATDA | 351 | YQNQR |
| 315 | VITVPNK | 352 | YSAY |
| 316 | VMK | 353 | YSF |
| 317 | VMPVQIAK | 354 | YSK |
| 318 | VNN | 355 | YSL |
| 319 | VQGGC | 356 | YSNK |
| 320 | VQNDK | 357 | YSTVQAQSGW |
| 321 | VSDSCGQ | 358 | YTEDEE |
| 322 | VSQHGINR | 359 | YVR |
| 323 | VTA | 360 | YVRPGLTP |
| 324 | VTEHK | 361 | YVTTS |
| 325 | VTSPCCIVTSQYGWSANMER | 362 | YWTA |
| 326 | VTSSCMNNGDSTGK | 363 | YYTSQSGDEVTS |
| 327 | VVGK |  |  |
| 328 | VVK |  |  |
| 329 | VVL |  |  |

**Table S2 Overview of Human Keap1 Protein PDB Files**

| **PDB ID** | **Keap1 Protein Region** | **Corresponding Full Keap1 Region** | **Structural Analysis Method** |
| --- | --- | --- | --- |
| 4CXT | Amino Acid Residues (49-180) | POZ Region | X-Ray Diffraction |
| 4CXI | Amino Acid Residues（50-179） | POZ Region | X-Ray Diffraction |
| 4CXJ | Amino Acid Residues（49-179） | POZ Region | X-Ray Diffraction |
| 4IFJ | Amino Acid Residues（322-609） | Kelch Region | X-Ray Diffraction |
| 4IFN | Amino Acid Residues（325-609） | Kelch Region | X-Ray Diffraction |
| 6SP4 | Amino Acid Residues（326-609） | Kelch Region | X-Ray Diffraction |
| 1U6D | Amino Acid Residues（322-609） | Kelch Region | X-Ray Diffraction |
| 6SP1 | Amino Acid Residues（325-609） | Kelch Region | X-Ray Diffraction |
| 7K2F | Amino Acid Residues（325-609） | Kelch Region | X-Ray Diffraction |
| 7K2G | Amino Acid Residues（327-614） | Kelch Region | X-Ray Diffraction |
| 7K2H | Amino Acid Residues（327-614） | Kelch Region | X-Ray Diffraction |
| 7K2I | Amino Acid Residues（325-609） | Kelch Region | X-Ray Diffraction |
| 7K2J | Amino Acid Residues（327-614） | Kelch Region | X-Ray Diffraction |
| 7K2L | Amino Acid Residues（326-614） | Kelch Region | X-Ray Diffraction |
| 7K2M | Amino Acid Residues（326-614） | Kelch Region | X-Ray Diffraction |
| 7K2N | Amino Acid Residues（327-609） | Kelch Region | X-Ray Diffraction |
| 7K2O | Amino Acid Residues（327-609） | Kelch Region | X-Ray Diffraction |
| 7K2P | Amino Acid Residues（327-609） | Kelch Region | X-Ray Diffraction |

| **PDB ID** | **Keap1 Protein Region** | **Corresponding Full Keap1 Region** | **Structural Analysis Method** |
| --- | --- | --- | --- |
| 7K2R | Amino Acid Residues（326-614） | Kelch Region | X-Ray Diffraction |
| 7K2S | Amino Acid Residues（326-614） | Kelch Region | X-Ray Diffraction |
| 5WFL | Amino Acid Residues（327-609） | Kelch Region | X-Ray Diffraction |
| 5WG1 | Amino Acid Residues（330-609） | Kelch Region | X-Ray Diffraction |
| 5WHL | Amino Acid Residues（330-609） | Kelch Region | X-Ray Diffraction |
| 5WHO | Amino Acid Residues（325-613） | Kelch Region | X-Ray Diffraction |
| 5WIY | Amino Acid Residues（326-609） | Kelch Region | X-Ray Diffraction |
| 7Q5H | Amino Acid Residues（325-609） | Kelch Region | X-Ray Diffraction |
| 7Q6Q | Amino Acid Residues（325-609） | Kelch Region | X-Ray Diffraction |
| 7Q6S | Amino Acid Residues（325-609） | Kelch Region | X-Ray Diffraction |
| 7Q8R | Amino Acid Residues（325-609） | Kelch Region | X-Ray Diffraction |
| 7Q96 | Amino Acid Residues（325-609） | Kelch Region | X-Ray Diffraction |
| 3ZGD | Amino Acid Residues（325-609） | Kelch Region | X-Ray Diffraction |
| 6FMP | Amino Acid Residues（325-609） | Kelch Region | X-Ray Diffraction |
| 6FMQ | Amino Acid Residues（325-609） | Kelch Region | X-Ray Diffraction |
| 6V6Z | Amino Acid Residues（325-609） | Kelch Region | X-Ray Diffraction |
| 1ZGK | Amino Acid Residues（322-609） | Kelch Region | X-Ray Diffraction |
| 4IN4 | Amino Acid Residues（322-609） | Kelch Region | X-Ray Diffraction |
| 4IQK | Amino Acid Residues（325-609） | Kelch Region | X-Ray Diffraction |
| 5DAD | Amino Acid Residues（50-179） | POZ Region | X-Ray Diffraction |

| **PDB ID** | **Keap1 Protein Region** | **Corresponding Full Keap1 Region** | **Structural Analysis Method** |
| --- | --- | --- | --- |
| 6TYM | Amino Acid Residues（325-609） | Kelch Region | X-Ray Diffraction |
| 6T7Z | Amino Acid Residues（326-609） | Kelch Region | X-Ray Diffraction |
| 7EXI | Amino Acid Residues（50-179） | POZ Region | X-Ray Diffraction |
| 7X4W | Amino Acid Residues（50-176） | POZ Region | X-Ray Diffraction |
| 7X4X | Amino Acid Residues（50-177） | POZ Region | X-Ray Diffraction |
| 6HWS | Amino Acid Residues（325-609） | Kelch Region | X-Ray Diffraction |
| 6UFO | Amino Acid Residues（324-609） | Kelch Region | X-Ray Diffraction |
| 4L7B | Amino Acid Residues（322-612） | Kelch Region | X-Ray Diffraction |
| 4L7C | Amino Acid Residues（326-611） | Kelch Region | X-Ray Diffraction |
| 4L7D | Amino Acid Residues（326-611） | Kelch Region | X-Ray Diffraction |
| 4N1B | Amino Acid Residues（326-611） | Kelch Region | X-Ray Diffraction |
| 5X54 | Amino Acid Residues（325-609） | Kelch Region | X-Ray Diffraction |
| 6TGB | Amino Acid Residues（322-609） | Kelch Region | X-Ray Diffraction |
| 3VNH | Amino Acid Residues（1-288） | NTR，POZ与IVR Region | X-Ray Diffraction |
| 7XM2 | Amino Acid Residues（325-609） | Kelch Region | X-Ray Diffraction |
| 3VNG | Amino Acid Residues（1-288） | NTR，POZ与IVR Region | X-Ray Diffraction |
| 7XM3 | Amino Acid Residues（323-609） | Kelch Region | X-Ray Diffraction |
| 7XM4 | Amino Acid Residues（325-609） | Kelch Region | X-Ray Diffraction |
| 7XM5 | Amino Acid Residues（324-609） | Kelch Region | X-Ray Diffraction |
| 6ROG | Amino Acid Residues（324-609） | Kelch Region | X-Ray Diffraction |

| **PDB ID** | **Keap1 Protein Region** | **Corresponding Full Keap1 Region** | **Structural Analysis Method** |
| --- | --- | --- | --- |
| 7K28 | Amino Acid Residues（326-609） | Kelch Region | X-Ray Diffraction |
| 7K29 | Amino Acid Residues（327-609） | Kelch Region | X-Ray Diffraction |
| 7K2A | Amino Acid Residues（327-609） | Kelch Region | X-Ray Diffraction |
| 7K2B | Amino Acid Residues（327-612） | Kelch Region | X-Ray Diffraction |
| 7K2C | Amino Acid Residues（327-609） | Kelch Region | X-Ray Diffraction |
| 7K2D | Amino Acid Residues（327-609） | Kelch Region | X-Ray Diffraction |
| 7K2E | Amino Acid Residues（328-609） | Kelch Region | X-Ray Diffraction |
| 7K2K | Amino Acid Residues（327-614） | Kelch Region | X-Ray Diffraction |
| 6FFM | Amino Acid Residues（50-180） | POZ Region | X-Ray Diffraction |
| 2FLU | Amino Acid Residues（325-609） | Kelch Region | X-Ray Diffraction |
| 5WFV | Amino Acid Residues（326-609） | Kelch Region | X-Ray Diffraction |
| 4XMB | Amino Acid Residues（324-609） | Kelch Region | X-Ray Diffraction |
| 3ZGC | Amino Acid Residues（325-609） | Kelch Region | X-Ray Diffraction |
| 6T7V | Amino Acid Residues（325-609） | Kelch Region | X-Ray Diffraction |
| 5GIT | Amino Acid Residues（51-180） | POZ Region | X-Ray Diffraction |
| 6W67 | Amino Acid Residues（51-179） | POZ Region | X-Ray Diffraction |
| 6W68 | Amino Acid Residues（51-179） | POZ Region | X-Ray Diffraction |
| 6W69 | Amino Acid Residues（51-179） | POZ Region | X-Ray Diffraction |
| 5NLB | Amino Acid Residues（51-204） | POZ Region | X-Ray Diffraction |
| 2XN4 | Amino Acid Residues（306-591） | Kelch Region | X-Ray Diffraction |

| **PDB ID** | **Keap1 Protein Region** | **Corresponding Full Keap1 Region** | **Structural Analysis Method** |
| --- | --- | --- | --- |
| 7XPY | Amino Acid Residues（555-1084） | CTR Region | X-Ray Diffraction |
| 2VPJ | Amino Acid Residues（279-567） | Kelch Region | X-Ray Diffraction |
| 6W66 | Amino Acid Residues（2-160） | NTR and POZ Region | X-Ray Diffraction |
| 4ASC | Amino Acid Residues（314-621） | Kelch and CTR Region | X-Ray Diffraction |
| 5F72 | Amino Acid Residues（325-609） | Kelch Region | X-Ray Diffraction |
| 4CH9 | Amino Acid Residues（300-585） | Kelch Region | X-Ray Diffraction |
| 4CHB | Amino Acid Residues（306-591） | Kelch Region | X-Ray Diffraction |
| 6GY5 | Amino Acid Residues（317-601） | Kelch Region | X-Ray Diffraction |
| 6TGY | Amino Acid Residues（2-105） | NTR Region | Electron Microscopy |
| 6TH3 | Amino Acid Residues（2-105） | NTR Region | Electron Microscopy |
| 4AP2 | Amino Acid Residues（67-336） | POZ and IVR Region | X-Ray Diffraction |
| 4APF | Amino Acid Residues（67-333） | POZ and IVR Region | X-Ray Diffraction |
| 6WCQ | Amino Acid Residues（2-160） | NTR and POZ Region | Electron Microscopy |
| 3II7 | Amino Acid Residues（290-577） | Kelch Region | X-Ray Diffraction |
| 7VIJ | Amino Acid Residues（555-1083） | CTR Region | X-Ray Diffraction |
| 6Z6A | Amino Acid Residues（325-609） | Kelch Region | X-Ray Diffraction |
| 4IFL | Amino Acid Residues（325-609） | Kelch Region | X-Ray Diffraction |
| 5DAF | Amino Acid Residues（50-177） | POZ Region | X-Ray Diffraction |
| 6TYP | Amino Acid Residues（326-609） | Kelch Region | X-Ray Diffraction |
| 7K2Q | Amino Acid Residues（327-614） | Kelch Region | X-Ray Diffraction |

**Table S3 Keap1 proteins containing ligand molecules**

| **ID** | **Ligand structure** | **Resolution（Å）** |
| --- | --- | --- |
| 4IFN |  | 2.40A |
| 6SP4 |  | 2.59A |
| 6SP1 |  | 2.57 Å |
| 4CHB | EPEEPEADQH  / | 1.56 |
| 6GY5 | LGLPDLVAKYN | 1.09 |
| 7K2F | GAEETGE | 2.37 Å |
| 7K2H | GLY-ASP-PRO-GLU-THR-GLY-GLU | 2.09 |
| 7K2I | GAPETGE | 2.42 |
| 7K2J | GDPEAGE | 2.52 |
| 7K2L | BAL-NPETGE | 1.98 |
| 7K2M | GEPETGE | 2.02 |
| 7K2N | BAL DPET GE | 2.22 |
| 7K2O | ABU DPET GE | 2.11 |
| 7K2P | DAV DPET GE | 2.11 |
| 7K2Q | ACA-ASP-PRO-GLU-THR-GLY-GLU | 2.37 |
| 7K2R | B3A-ASP-PRO-GLU-THR-GLY-GLU | 2.10 |
| 7K2S | B3A-ASP-PRO-GLU-THR-GLY-GLU | 2.13 |
| 5WG1 | LDEEA GEFL | 2.20 |
| 5WHL |  | 2.50 |
| 5WHO |  | 2.23 |
| 5WIY |  | 2.23 Å |
| 7Q5H |  | 2.31 Å |
| 7Q6Q |  | 2.55 Å |
| 7Q6S |  | 2.14 Å |
| 7Q8R |  | 2.28 Å |
| 7Q96 |  | 2.42 Å |
| 6FMP | ACY-ASP-GLU-GLU-THR-GLY-GLU-PHE/ACE DEETGEF | 2.92 Å |
| 6FMQ | ACY-SC1-GLU-THR-GLY-GLU-LEU/ACE DYW ETGEL | 2.10 Å |
| 6V6Z |  | 1.60 Å |
| 1ZGK |  | 1.35 Å |
| 4IN4 |  | 2.59 |
| 4IQK |  | 1.97 |
| 6TYM |  | 1.42 |
| 6T7Z | ACY-SC1-ASA-4FB-GLU-THR-GLY-GLU | 2.00 |
| 6HWS |  | 1.75 |
| 6UFO |  | 2.68 Å |
| 4L7B |  | 2.41 Å |
| 4L7C |  | 2.40 Å |
| 4L7D |  | 2.25 Å |
| 4N1B |  | 2.55 Å |
| 5X54 | ACE-GLU-TRP-TRP-TRP/ACE EWWW | 2.30 Å |
| 6TG8 | VINPETGEQI Q | 2.75 |
| 7XM2 |  | 2.30 |
| 7XM3 |  | 2.80 |
| 7XM4 |  | 2.70 |
| 6Z6A |  | 2.37 Å |
| 4IFL | AFFAQLQLDEETGEFL | 1.80 Å |
| 7K28 | ADEETGEFL | 2.15 Å |
| 7K29 | ACE-LEU-ASP-GLU-GLU-THR-GLY-GLU-ALA-LEU-NH2/ACE LDEETGEAL NH2 | 2.20 Å |
| 7K2A | ACE-LEU-ASP-GLU-GLU-THR-GLY-GLU-PHE-ALA-NH2/ACE LDEETGEFA NH2 | 1.90 Å |
| 7K2B | ACE-ALA-ASP-GLU-GLU-THR-GLY-GLU-PHE-ALA-NH2/ACE ADEETGEFA NH2 | 2.31 Å |
| 7K2C | ACE ADEETGEAA NH2 | 2.11 Å |
| 7K2D | Ace-GDEETGE-NH2 | 2.21 Å |
| 7K2E | GLY-ASP-GLU-GLU-THR-GLY-GLU GDEETGE | 2.03 Å |
| 7K2K | BAL-ASP-GLU-GLU-THR-GLY-GLU | 1.98 Å |
| 2FLU | AFFAQLQLDEETGEFL | 1.50 Å |
| 5WFV | LDEETGEFL | 1.91 Å |
| 4XMB |  | 2.43 Å |
| 3ZGC | GDEETGE | 2.20 Å |
| 6T7V | LDPETGEFL | 2.60 Å |
| 4ASC |  | 1.78 Å |
| 4CH9 | EPEEPEADQ | 1.84 |
